# Successful cryopreservation of marine invertebrates immune cells enables long-term studies of common octopus, *Octopus vulgaris* Cuvier 1797, hemocyte immune functions

**DOI:** 10.3389/fimmu.2025.1543587

**Published:** 2025-03-21

**Authors:** María M. Costa, Estefania Paredes, Mercedes Peleteiro, Lucía Sánchez-Ruiloba, Francisco Gambón, Sonia Dios, Camino Gestal

**Affiliations:** ^1^ Instituto de Investigaciones Marinas (IIM), CSIC, Vigo, Spain; ^2^ Coastal Ecology Research Group (ECOCOST), Department of Ecology and Animal Biology, Marine Research Center, University of Vigo, Vigo, Spain; ^3^ CINBIO, Universidade de Vigo, Vigo, Spain; ^4^ Department of Immunology, Vigo University Hospital Complex, Vigo, Spain

**Keywords:** MAS medium, acclimation, optimal thawing, functional activity, cell viability, phagocytosis, invertebrate immunity

## Abstract

The common octopus, *Octopus vulgaris* Cuvier 1797, as all cephalopods, presents highly evolved characteristics compared to other classes of molluscs and the whole invertebrate phyla. However, to date, there is not much information about its immune system, and studying the defense mechanisms is a key step in understanding their response to external aggressions, having the tools to anticipate animal health problems and ensure their welfare. The lack of cell cultures in molluscs is a major problem when carrying out *in vitro* assays that help to deepen our knowledge of this species’ main immune cells. Cryopreservation becomes an alternative to maintaining viable and functional cells after freezing/thawing processes. Having access to good high-quality cells for long periods allows cover a wider repertoire of studies, time courses, and the avoidance of logistical issues such as loss of viability and/or functionality, time constraints, or sample transport challenges. Additionally, high-quality cell suspensions are essential for successful applications, such as single-cell sequencing, where viability and functionality are the key to optimal identification. The optimal medium, cryoprotective agent, and freezing/thawing protocol for octopus hemocytes have been selected. We show here the first functional results from cryopreserved hemocytes. Cells cryopreserved in MAS medium supplemented with EG maintained viability above 80% after 15 weeks post cryopreservation storage at -80°C, and their functional ability to phagocytize bacteria similar to fresh cells. Moreover, thawed acclimated cells exhibited a gene expression pattern comparable to fresh cells, as opposed to directly thawed cells. The acclimation process after thawing was essential to recover the functional activity of the cells and to return to levels of gene expression involved in oxidative stress similar to fresh cells.The results presented here will facilitate functional studies of octopus immune cells and provide tools for cell preservation in other molluscs species.

## 1 Introduction

Cephalopods in general, and common octopus, *Octopus vulgaris* Cuvier 1797, in particular, have been considered an enigmatic group of animals that have evolved to acquire remarkable capacities representing the most advanced class of the phylum Mollusca and showing innovative traits (1). The presence of highly evolved features (propulsion movements, camouflage cells (chromatophores), excellent vision, nervous system with learning capacity, or the presence of a wide neuronal network with a very complex brain) has made it an excellent model species for multiple physiological and neurological biomedical studies (2), as well as for behavioral research (3, 4).

As with their brain, nervous system, and other physiological components, the immune system of these organisms, despite being an innate immune system as invertebrates, may present more advanced characteristics than those of other related species. Cephalopods possess a close and well-developed circulatory system composed of veins and arteries. The hemocytes, the circulating cells of the hemolymph, are involved in several functions, being the principal cells of the immune system. The identification of highly polymorphic molecules in these animals (5) could also be a mechanism established within the immune system to enhance pathogen detection and enable a more effective immune response. This makes the study of cephalopod immunology particularly significant, as understanding their immune response can offer valuable insights not only into their biology but also into fundamental and comparative immunology. Their privileged phylogenetic position as the most advanced invertebrates further establishes them as an excellent model for such research. However, the knowledge of the immune system of cephalopods is nowadays limited. Further studies and the identification of the molecular and functional mechanisms that regulate their defense system will allow to advance into the knowledge of this species. The application of hemocytes massive sequencing technologies has offered novel precious information suggesting that a complex immune response might be operating in these animals (5–10). However, additional studies to deep into the knowledge of their immune system are needed.

Knowing the mechanisms by which this species immune system recognizes foreign organisms and triggers a response against them, becomes one of the key points to understand how its defense system works. It is important to note that the octopus, as an invertebrate animal, does not possess an adaptive immune system, capable of creating a secondary response based on the production of antibodies and adaptive memory, but simply possesses an innate system that constitutes its first line of defense against pathogens and microorganisms (7). This system, although more primitive at the phylogenetic level, has highly efficient strategies, from the simplest mechanisms such as the production of toxic oxygen or nitrogen radicals (11), to the recognition of pathogen-associated molecular patterns (6) and the triggering of cell signaling pathways that ultimately activate the expression of specific effector genes (5–10). Although these mechanisms have already been described in hemocytes of the common octopus and several effector and modulatory genes have been reported, it is necessary to carry out in-depth studies to understand each cell type (both at the molecular and functional level) and to be able to explore these functions in detail. In this sense, single-cell RNA sequencing (scRNA-seq) analysis becomes a powerful tool that allows the study of gene expression at the single-cell level (12). Unlike traditional RNA sequencing methods that analyze bulk populations of cells, scRNA-seq provides much higher resolution, revealing cell heterogeneity within a sample (13). However, to successfully perform scRNA-seq analysis with reproducible and high-quality results, it is crucial that the starting cell population strictly meets several requirements such as; high cell viability (as dead or damaged cells can release degraded RNA that affects data quality); high number of cells to ensure adequate and representative coverage of the cell population, and high quality of the cell population where the cells are completely dissociated, without the presence of aggregates and in a free of contaminants and cell debris medium that respects their integrity. Moreover, cells must be carefully handled during all steps of the process to ensure their functional capabilities and minimize cell death and stress, which could potentially alter gene expression profiles, so the choice of medium becomes another fundamental requirement of this procedure (14).

Consequently, having an inexhaustible source of biological material from cell cultures could be a valuable tool; not only to facilitate laboratory tasks and increase biological replication, but also as a replacement technique to contribute to the achievement of the 3Rs goal in animal experimentation (15). However, investigating cephalopod immune cells presents significant challenges, primarily due to the lack of immune cell lines in molluscs, to the difficulty of maintaining viable samples in primary cultures, and to the absence of standardized methodologies. One of the main obstacles to conducting functional and molecular studies on molluscs cells is the absence of immortal cell lines. Although primary cultures have been achieved in some species up to 40 days old (16–18) no established hemocyte cell line has been developed to date. While primary neuron cultures have been successfully developed for this species (19), no reports exist on the culture of immune cells, the only one being those published by Necco and Martin (20), who were able to maintain hemocytes obtained from the white body under mitotic divisions, although for no longer than 24 h. The availability of cell culture tools would allow less limited studies to be carried out, where uniformity between replicates, adequate sample size or continuous availability of replicates would greatly facilitate the experimental design of many assays. Additionally, it must be considered that these cells cannot withstand long periods in primary cultures as one of the defense strategies of these invertebrate organisms is based on the capacity of aggregation of their main phagocytic cells: the hemocytes. This often renders long-term assays unfeasible. Moreover, for many techniques, the use of individual cells is recommended or mandatory (e.g., flow cytometry and scRNA-seq) (14). Maintaining cells in primary culture increases the likelihood of aggregations, even when using anti-aggregation solutions, and makes it difficult to carry out this type of technique without interferences. Therefore, in the absence of cell cultures that keep cells viable and individualized for long periods, it is necessary to design other strategies that allow the analysis of a wide range of cell parameters in a time-efficient and feasible manner.

One promising approach to overcoming these obstacles is cryopreservation, which provides a potential solution for long-term storage and analysis of immune cells while preserving their functionality. Therefore, it stands out as an excellent alternative for freezing and thawing cells while maintaining their integrity and viability. Although several invertebrate marine species have already standardized cryopreservation protocols for sperm cells or even for whole embryos and larvae (21–24), a unified protocol is not available for different cell or tissue types. Instead, rather each step of the process needs to be optimized, from the choice of cryoprotectant agent (CPA) and the study of its toxicity effects to the freezing/thawing conditions. The high diversity of marine species and their differences in shape, complexity, size, or even in living/feeding/reproducing mechanisms, make this process extremely meticulous, requiring detailed studies of each stage, with a high risk of loss of cell viability if any step of the process is not fully optimized. Even in cells from the same organism, conditions can vary radically and the same CPA or freezing/thawing method can be toxic or lethal for some cells and not for others (25, 26). Additionally, some cell types are known for being particularly complicated to cryopreserve. For instance, in the field of marine biology, there are multiple studies on sperm cryopreservation in different species, while the process of preserving oocytes has not yet been successfully developed.

The use, study, and culture of marine invertebrates have a wide range of applications. These include contributions from biomedical, physiological, ecological, toxicological, and socioeconomic perspectives (27). As such, the potential applications of cryopreservation are as diverse as the applications of each of the species. In aquaculture, cryopreservation has already played a pivotal role in the possible application of selective breeding programs, preserving of biodiversity through biobanks, and improving hatchery spat overcoming the limitations of this important industry (28, 29). Despite the broad potential of this field, to our knowledge, there has yet to be an optimized cryopreservation protocol for immune cells in any marine invertebrate species. This gap makes it difficult to study their identification, both molecular and functional, and keeps closed a door that would allow many studies that, to date, have not been possible.

In the current paper, we present the first results of long-term storage of octopus hemocytes. To our knowledge, this represents the first successful cryopreservation of marine invertebrate immune cells. These results open up a wide range of opportunities for the study of these key cells in the defense system of these organisms. This study aims to address a critical knowledge gap in cephalopod immunology by providing insights into effective cryopreservation techniques and their implications for immunological research. Cryopreservation not only offers a method for long-term storage but also serves as a valuable alternative for conducting *in vitro* assays, enabling experiments at different time points, ensuring reproducibility, and reducing the need for live animal experimentation, thereby promoting more ethical and sustainable research practices. Maintaining a source of viable, functional hemocytes allows for experimental assays that would otherwise be impossible to complete in a single day, expanding the range of variables and sampling times while also facilitating inter-laboratory analyses. The relevance of cryopreservation is further emphasized by the absence of established primary cultures or cell lines for *Octopus vulgaris* hemocytes. This limitation underscores the crucial role of cryopreservation in cephalopod immunology, as it currently represents the only viable method for maintaining immune cells. It is also important to note that the ability to preserve these cells in an active functional state will also help lay the groundwork for the development of potential future cell lines, which would be a significant advancement in invertebrate immunology methodologies.

This tool will offer substantial advantages for studying the most important cells involved in pathogen defense in *O. vulgaris*, enhancing our understanding of the defense mechanisms of this species. Given the enormous potential both as a model species and candidate for aquaculture diversification, this research will lead to implementing improvements in their culture conditions and supporting the development of strategies to preserve its health and welfare.

## 2 Materials and methods

### 2.1 Animal sampling and care

A total of four specimens of *O. vulgaris* (measuring 1.5 kg on average) were collected by traps, an artisanal fishing gear used by local fishermen from the Ria of Vigo, Spain (24° 14.09′N, 8° 47.18′W). Animals were properly transported to the Experimental Culture Facilities of IIM-CSIC (center for the breeding and use of experimental animals under the REGA code ES360570202001). Individuals were maintained in 500 L tanks of filtered aerated seawater at 15 ± 1°C with a continuous re-circulating flow. The photoperiod was 12 h light:12 h dark and cleaning and parameter checks were performed daily. They were fed daily with frozen fish and fresh mussels. Before the experimental trial, octopuses were acclimated at least for one week. All animals were individually kept due to their territorial and cannibal nature.

### 2.2 Ethical considerations

Transport, housing, handling, and experimentation were performed according to the Spanish law RD53/2013 within the framework of the European Union directive on animal welfare (Directive 2010/63/EU) for the protection of animals employed for experimentation and other scientific purposes, following the Guidelines for the care and welfare of cephalopods published by Fiorito et al. (30). The animal study was reviewed and approved by the Ethic Committee of the National Competent Authority (ES360570202001/21/FUN.01/INM06/CGM01). All experiments conducted with these animals were performed by qualified and duly accredited personnel, ensuring compliance with the principles of the Three Rs (Replacement, Reduction, and Refinement) to promote ethical and responsible research practices.

### 2.3 Hemolymph collection

Animals were bath anesthetized by immersion in 3L of a 1.5% MgCl_2_ solution dissolved in filtered seawater (FSW) supplemented with 1% ethanol (VWR) (30). After hemolymph extraction, the animals were then returned to the tanks, with increased aeration for 15-30 minutes, to recover from the effects of the anesthesia. Hemolymph samples were collected from the caudal vein using a disposable syringe previously semi-filled with marine antiaggregant solution (MAS) (16) to dilute the hemolymph in a proportion 1:1 and avoid the cell aggregation of the collected hemocytes. A maximum of 1 mL hemolymph was extracted from each octopus, and repeated after at least 6 days, if additional hemolymph was needed for the experiments, according to (31). This protocol avoids variations in hemolymph properties and hemocyte number allowing to revert starting points before each sampling and maintaining the welfare (32). Samples were immediately centrifuged at low speed to get functional cells (300xg for 5 min at 4°C). Supernatants were separately placed in a fresh tube and centrifuged at maximum speed (13000xg) to obtain a cell free *O. vulgaris* serum (OS) as the medium for the next experiments. Cell pellets were resuspended to get a final concentration of 3x10^6^ cells/mL in each of the tested media described below and kept at 4°C.

### 2.4 Media and cryoprotectant agents

To evaluate the effect of different solutions on the cryopreservation process, serum from *O. vulgaris* (OS), MAS medium, Squid Ringer’s solution (SRS) (33), and RPMI 1640 (Gibco) medium were used to resuspend the cells. Three biological replicates of each cell suspension with a total of 3x10^6^ cells were used to test each candidate media. Two aliquots of each medium were used to check the effects of ethylene glycol (EG) (Scharlau) and dimethyl sulfoxide (DMSO) (Fisher Chemical) as CPAs. For that, one replicate was supplemented with a solution of 15% of EG and another with 10% of DMSO. The optimal doses for cell preservation for both CPAs were determined through dose-response assays, testing a range of concentrations and evaluating the effect on cell viability for DMSO (data not shown) and previous results by Lago et al. (34) for EG. The remained replicate was kept without any CPA and acted as negative control. All aliquots (with a total volume of 800 μL (400 μL of cells and 400 μL of CPA)) were placed in a Mr. Frosty™ freezing container previously cooled to 4°C and filled with isopropanol, and immediately placed at -80°C to obtain a slow and progressive freezing rate (Mr. Frosty produces a temperature decrease of approximately 1°C min^-1^). This experiment was repeated three times.

### 2.5 Thawing conditions and acclimation

To determine the optimal thawing protocol, two different methodologies (“slow or progressive” and “fast or non-progressive” conditions) were tested. For the “slow protocol”, cells were thawed in a water bath at 30°C. As soon as the sample showed the first signs of thawing, the original medium without CPA in which the cells were originally frozen was used to slowly dilute the cell suspension by up to two fold, alternating the addition of medium with gentle mixing movements. Cells were slowly transferred into a 50 mL tube (one drop every 5 seconds) and the medium was slowly and progressively diluted up to tenfold. The cells were washed twice by centrifugation at 300xg for 5 min at 4°C. The final pellet was resuspended in 400 µL of each tested CPA-free medium. For the “fast protocol” frozen vials were also placed in a water bath at 30°C and the cells were diluted rapidly and only to twice their volume with CPA-free medium. The cells were pelleted by centrifugation. The supernatant was removed and the cells were resuspended in a final volume of 400 µL of each tested CPA-free medium. This experiment was repeated at least three times.

After determining the optimal conditions for cell cryopreservation and thawing, which were established based on the assessment of cell viability as described in the results section, the impact of cell acclimation following the thawing process was assessed. To evaluate this, thawed cells were resuspended in an appropiate volume of the selected media, seeded in 8-well plates, and maintained at 15°C for 18h prior processing. Three experimental groups were analyzed using functional and molecular assays: i) fresh cells; ii) directly thawed cells (non-acclimated) and iii) thawed cells that underwent acclimatation (thawed acclimated cells).

### 2.6 Viability assays

A total volume of 100 µL of each set of cell suspension was incubated in the dark with 2 µL of propidium iodide (PI) (Invitrogen) (1mg/mL) or 0.5 µL of DRAQ7 (Biostatus) (0.3 mM) before measurement by flow cytometry using a Cytoflex (Beckham Coulter), in the Flow Cytometry Service at CINBIO (University of Vigo). PI and DRAQ7 are membrane-impermeable dyes that specifically stain the nuclei of dead cells, as they are excluded from viable cells. Upon excitation by specific lasers (560 nm for PI and 640 nm for DRAQ7), these dyes emit fluorescence at wavelengths of 617 nm (PI) and 665 nm (DRAQ7) when bound to double-stranded DNA. This fluorescence is detected by the corresponding channels and correlates with the percentage of dead cells. To select the optimal medium and CPA, and prior to the analysis of the acclimation effect, PI was used for the initial quantification of the viability after the freezing/thawing process for fresh and directly thawed cells. For the following flow cytometry viability experiments described, DRAQ7 was used instead of PI because of its compatibility to be visualized at the same time in the fluorescent microscopic analyses. To further analyze the effects of cell acclimation after thawing, all three experimental groups (fresh, directly thawed, and thawed acclimated cells) were analyzed. For the thawed acclimated set, cells of each thawed vial were resuspended after thawing in a total of 300 µL of the selected media, seeded in an 8-well chamber slide, and kept at 15°C for 18h before processing. The negative fluorescence threshold and possible autofluorescence emission of hemocytes were previously established and tested using unlabeled cells. This experiment was repeated at least three times.

Fluorescence microscopy was performed to visualize cell morphology and viability following the freezing and thawing protocol. A batch of cells was directly thawed following the optimal protocol previously described and seeded into an 8-well plate in a final volume of 300 µL of MAS media. Fresh cells were seeded under identical conditions. To analyze the possible impacts of the freezing/thawing protocol on the cell morphology, the last experimental group (thawed acclimated cells) was also seeded and kept in MAS media for a total of 18h before the analysis and maintained at 15°C before the image analysis. The morphology of fresh, directly thawed, and thawed acclimated cells was analyzed under a Thunder fluorescent inverted microscope (Leica). Images were recorded for 4h every 10 minutes and analyzed to detect morphology differences for the different cell types. This experiment was repeated three times.

### 2.7 Phagocytic assays

Once the optimal medium, CPA, and thawing protocol (based on the cell viability and microscopy morphology results) were selected, cells were frozen and thawed using the optimal conditions. The phagocytic activity of fresh and directly thawed cells was determined by flow cytometry and by fluorescent microscopy, measuring the ability of the hemocytes to phagocytize a fluorescent and heat-killed inactivated bacteria (*Vibrio lentus*, CECT 5110T, kindly provided by Dr. Rosa Farto from University of Vigo). For flow cytometry analysis, octopus hemocytes were dispensed into 96-well plates (100 μL per well) and, after 30 min of incubation at 15°C, a solution of 100 μL of FITC-labeled bacteria with a concentration adjusted to 1:10 (cells:bacteria) proportion was added to the cells. Control cells were incubated with the same volume of FSW. After incubation in the dark at 15°C for 2 h, the non-internalized particles were removed by washing twice with 100 μL of FSW. The attached cells were finally resuspended in 200 μL of FSW. Twenty microliters of 0.4% trypan blue solution in FSW were added to each sample to quench the fluorescence of adhered but non-phagocyted bacteria. Cells were analyzed in a Cytoflex flow cytometer (Beckham Coulter), in the Flow Cytometry Service at CINBIO (University of Vigo). Phagocytosis was analyzed and expressed as the percentage of cells that have internalized fluorescent bacteria (positive cells) and as the median of the fluorescence intensity, which indicated the quantity of fluorescent bacteria phagocyted by each cell. Fresh cells were used as the positive control. To analyze the reduction of the possible stressful effect of the freezing/thawing protocol on the cell response to fluorescent bacteria, a new set of cells was acclimated to the media after thawing (thawed acclimated cells) for 18h by seeding into a 96-well plate and kept at 15°C in the dark before the evaluation of their phagocytic activity. This experiment was repeated at least three times.

The phagocytic activity was also analyzed under the Thunder fluorescence inverted microscopy for the three experimental groups (fresh, directly thawed, and thawed acclimated cells). A total volume of 100-200 µL of each cell suspension was seeded into an 8-well plate in the presence or absence of a FITC fluorescent labeled bacteria, which was added to the cells in a proportion of 1:10 (cell:bacteria). Images were recorded for 4h every 10 minutes. Image analysis was performed to detect differences in the functional activity of the different cell types.

### 2.8 Molecular assays

**Figure 1:**
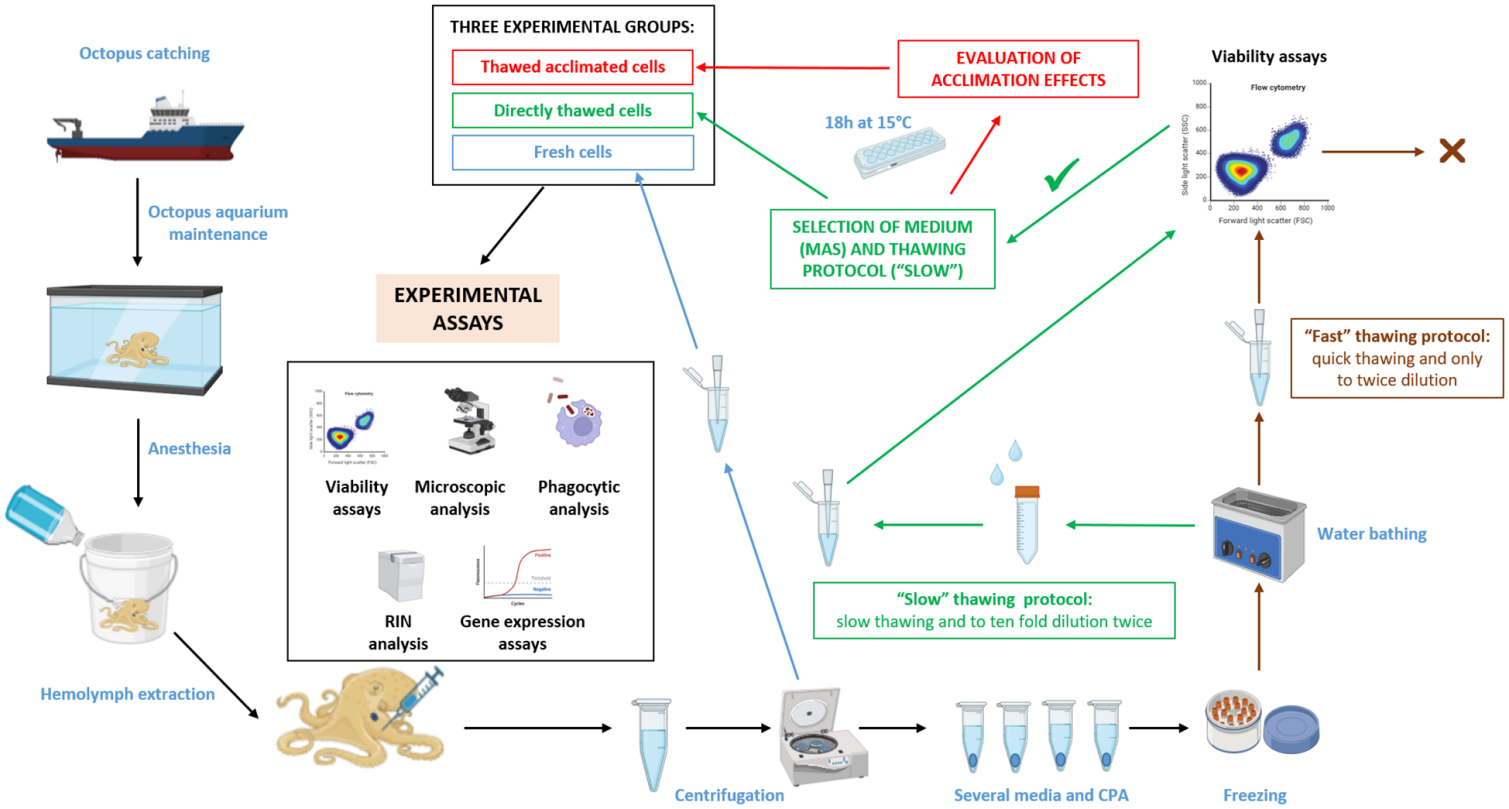
Workflow used for selecting a cryopreservation method of octopus hemocytes and subsequent evaluation of hemocytes functional activities. Partially created with BioRender.com.

To quantify the effect of the optimized freezing/thawing protocol on the RNA quality and gene expression efficiency, total RNA from cryopreserved cells was isolated and compared with total RNA directly isolated from frozen samples where cells were kept directly in TRI Reagent^®^ (MRC). RNA purification was performed with the Direct-zol RNA Miniprep Kit (Zymo Research), which included a DNase I treatment following the manufacturer’s instructions. The quality of isolated RNAs was tested using a Bioanalyzer (Agilent). A total of 1000 ng of RNA was used to be reverse transcripted to cDNA using the Maxima First Strand cDNA Synthesis Kit for RT-qPCR (ThermoFisher). Octopus ubiquitin expression was used as housekeeping gene (35), to check the reverse transcription efficiency. Quantitative PCR (qPCR) assays were performed using Quant Studio 3 (Applied Biosystems). A total volume of 25 μL PCR mixture included 12.5 μL of SYBR Green PCR master mix (Applied Biosystems), 0.5 μL of each primers pair 10 μM, and 1 μL of cDNA. Amplification was carried out at the standard cycling conditions of 95°C for 10 min, followed by 40 cycles of 95°C for 15 s and 60°C for 1 min. The expression of stress-related genes (superoxide dismutase (SOD), catalase (CAT), and glutathione peroxidase (GPX)) was evaluated in fresh, directly thawed, and thawed acclimated cells. The comparative Ct method (2-ΔCt method) was used to determine the expression level of analysed genes (36). The expression of the candidate genes was normalized using ubiquitin as housekeeping gene. Each reaction was conducted in triplicate and a total of five biological replicates were used for each analysis. The primer sequences can be found in **Table 1**. To better understand the process, a chart of methodology workflow is shown in **Figure 1**.

**Table 1:** List of primers used for the molecular expression analysis.

Primer name	Sequence (5’-3’)	Amplified gene	Reference
Ubiquitin-OV-qPCR F	AGAAGGTTAAGTTGGCGGTTTTG	Ubiquitin	García-Fernández et al. (35)
Ubiquitin-OV-qPCR R	CCAGCTCCACATTCCTCGTT		
QPCR CAT-F	TGCAAGTGACCCAGATTATG	Catalase	Authors design (SRA; PRJNA547720)
QPCR CAT-R	TGACTGGTAGCTGGAGATAC		
QPCR GPX7-F	TGCCAGTGAATGTGGTTAC	Gluthatione Peroxidase	Authors design (SRA; PRJNA547720)
QPCR GPX7-R	AGAAGTTGGTAGCGTTTGG		
SOD F3 Ov	TTGCTACACCCTGACCAGAG	Superoxide Dismutase	Authors design (SRA; PRJNA547720)
SOD R3 Ov	TGGACATTTCAACCCAGAGA		

### 2.9 Image analysis

The images were analyzed using Zeiss Arivis Pro software (Version 4.2, Carl Zeiss Microscopy GmbH). Quantification was carried out using a pipeline (a predefined sequence of commands) that allows the segmentation of the cells in the brightfield channel. Each segmented cell was classified as positive or negative for intracellular fluorescence signal if fluorescence intensity was above the threshold previously determined in the pipeline. Multiple images were analyzed per condition to ensure statistical robustness.

### 2.10 Statistics

Viability results were analyzed using a three-way ANOVA with medium, type of CPA, and thawing method as fixed factors. Before analyses, normality and homogeneity of variances were checked by Shapiro-Wilk and Levene’s tests, respectively. In case the homogeneity of variances is violated, raw or rank-transformed data were considered to run the ANOVAs (37). Results were considered significant at p ≤ 0.05. The univariate test of significance was used to elucidate how much of the total variation in the dependent variable can be attributed to differences between factor levels and to know which of the factors have a stronger effect on the dependent variable. Homogeneous groups were established *a posteriori* with Tukey test for multiple comparisons. For fluorescence mean comparisons, Student t-test was used. Statistical analysis was done using the STATISTICA v.7.0 software (StatSoft). Data are reported as means + SEM.

## 3 Results

### 3.1 Screening of the optimal medium and conditions for *O. vulgaris* hemocytes cryopreservation

**Figure 2:**
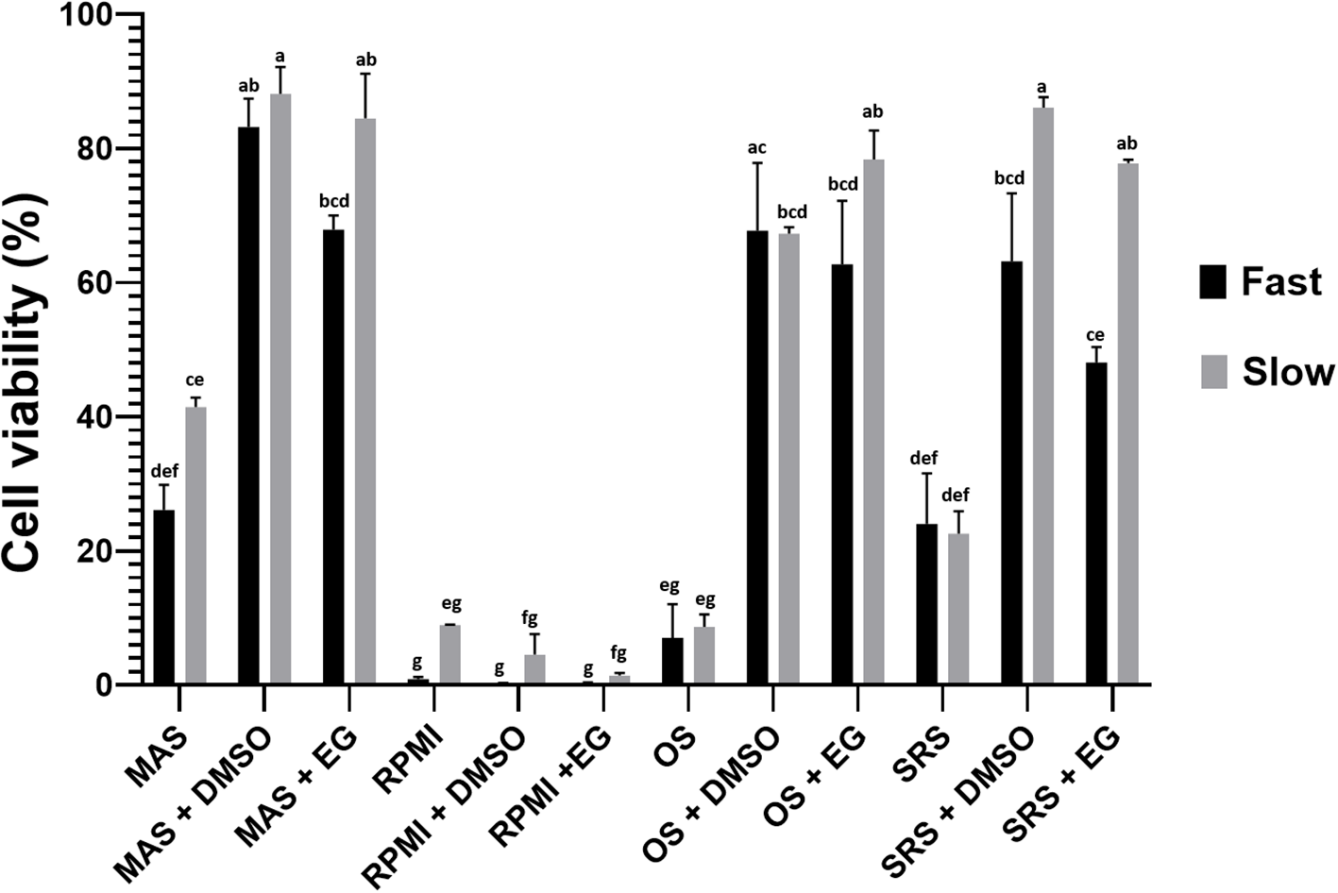
Percentage of cell viability (hemocytes) after cryopreservation with the different tested media and thawing following the two evaluated methods. Data represent the mean of values obtained in each experiment + SEM. The letters indicate statistical comparisons among the 24 experimental groups, where bars sharing the same letter do not present significant differences, while bars with different letters indicate statistically significant differences (p ≤ 0.05).

Different percentages of viability were found for each analyzed condition (**Figure 2**). The experimental factors —culture medium, thawing methodology, and presence or absence of cryoprotective agents (CPA)— were statistically significant both individually and in combination (**Table 2**). According to our statistical analysis, the choice of medium for the freezing/thawing process emerged as the primary determinant of cell viability. Cells preserved in MAS exhibited the highest viability rate, even under “fast” freezing/thawing conditions, surpassing those presenting higher values of cell viability than other media used in “slow” conditions. Conversely, cells kept on RPMI medium showed the lowest viability regardless of the freezing/thawing method or the CPA used. The presence of a CPA in the freezing medium was also crucial for the cells, ranking as the second most influential factor for cell viability. In most media, viability rates were higher when the CPA was present, except in the case of RPMI, where deleterious effects of the medium itself were so pronounced that CPAs failed to mitigate the damage. No significant differences were noted between DMSO and EG as CPAs across media and freezing/thawing conditions. The thawing method ranked third in statistical significance, with notable differences between “slow” and “fast” thawing observed only for cells in RPMI and SRS medium.

**Table 2:** Summary of the univariate multivariate significance tests used in the multifactorial ANOVA.

		Sum of squares (SS)	Degrees of Freedom (DF)	Mean Square (MS)	F statistics	p value
**Variables**	**Intercept**	28812.00	1	28812.00	2930.034	**0.000000**
	**Thawing**	408.33	1	408.33	41.525	**0.000001**
	**Media**	5251.17	**3**	**1750.39**	**178.006**	**0.000000**
	**CPA**	1828.63	2	914.31	92.981	**0.000000**
	Thawing*Media	73.17	3	24.39	2.480	0.085391
	Thawing*CPA	91.79	2	45.90	4.667	**0.019399**
	Media*CPA	1174.71	6	195.78	19.910	**0.000000**
	Thawing*Media*CPA	148.21	6	24.70	2.512	**0.049724**
	**Error**	236.00	24	9.83		

Bold F values indicate the strength of group differences in ANOVA; higher values suggest stronger effects. Significance is determined by p-values (p ≤ 0.05). The shading intensity reflects the strength of each factor's effect, based on the F statistic. Darker shades indicate higher F values and stronger effects, while lighter shades represent weaker effects.

According to these results, MAS medium was selected to be supplemented with CPA (we selected EG instead of DMSO for the reason elaborated in the subsequent section) and opted for “slow” thawing conditions for further experiments.

### 3.2 Cell survival over time and morphological aspect

**Figure 3:**
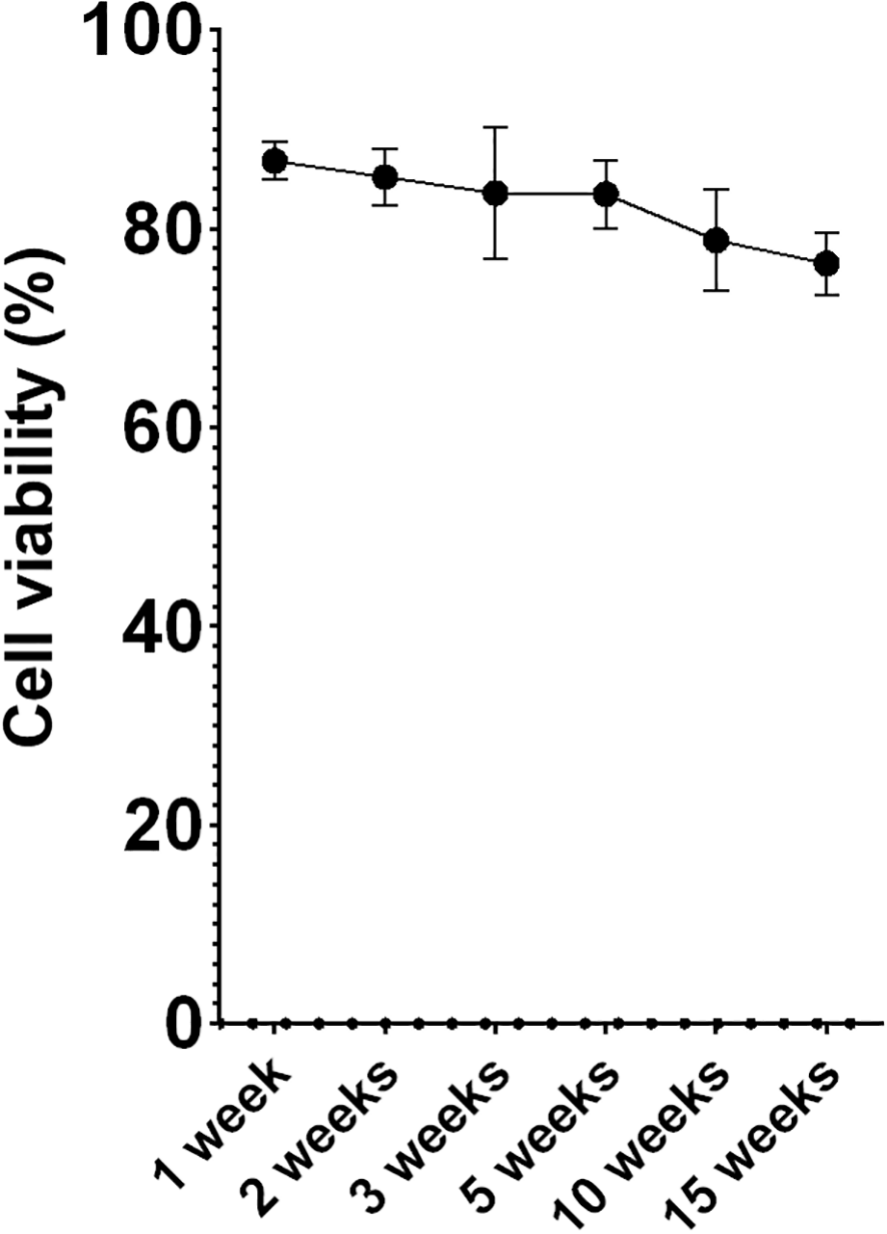
Percentage of cell survival (hemocytes) over time. Data represent the mean of values obtained in each experiment + SEM.

**Figure 4:**
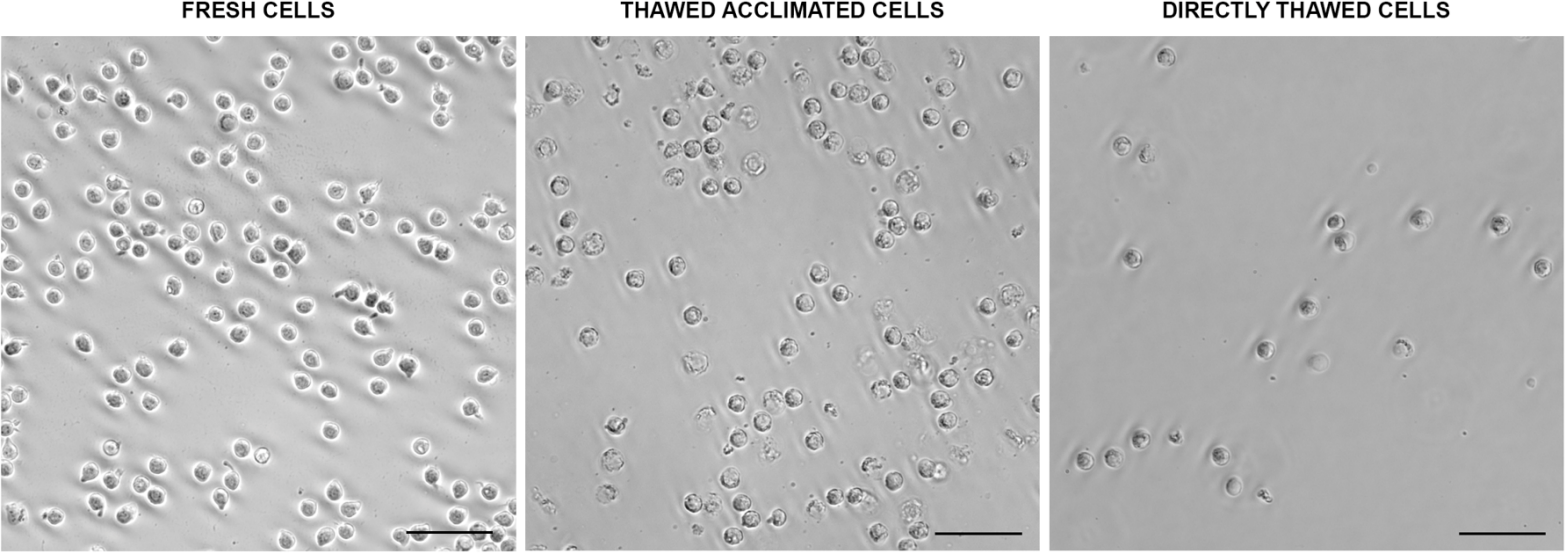
Morphological aspects of fresh, thawed acclimated, and directly thawed cells (hemocytes) visualized under the optical microscope. Scale bar: 50 µm.

Multiple aliquots were maintained at -80°C at different time points using MAS medium supplemented with EG. Following the optimized freezing/thawing protocol, cell viability was measured by flow cytometry. While viability values showed a slight decline over time, cells retained viability levels over 75% (**Figure 3**) after 15 weeks of storage, indicating that this protocol was highly effective for this cell type. Microscopic evaluation showed that the thawed cells required more time to produce pseudopodia compared to fresh cells. However, after an 18-hour acclimation period, thawed acclimated cells resembled to fresh cells, with cell membranes appearing less rigid than directly thawed cells. Additionally, acclimated cells were less round with their membranes beginning to produce some movements and invaginations (**Figure 4**), a sign of returning to normal cell dynamics, as observed in the recordings we made (data not shown), from which the images presented in this article were taken.

### 3.3 Functional assays

**Figure 5:**
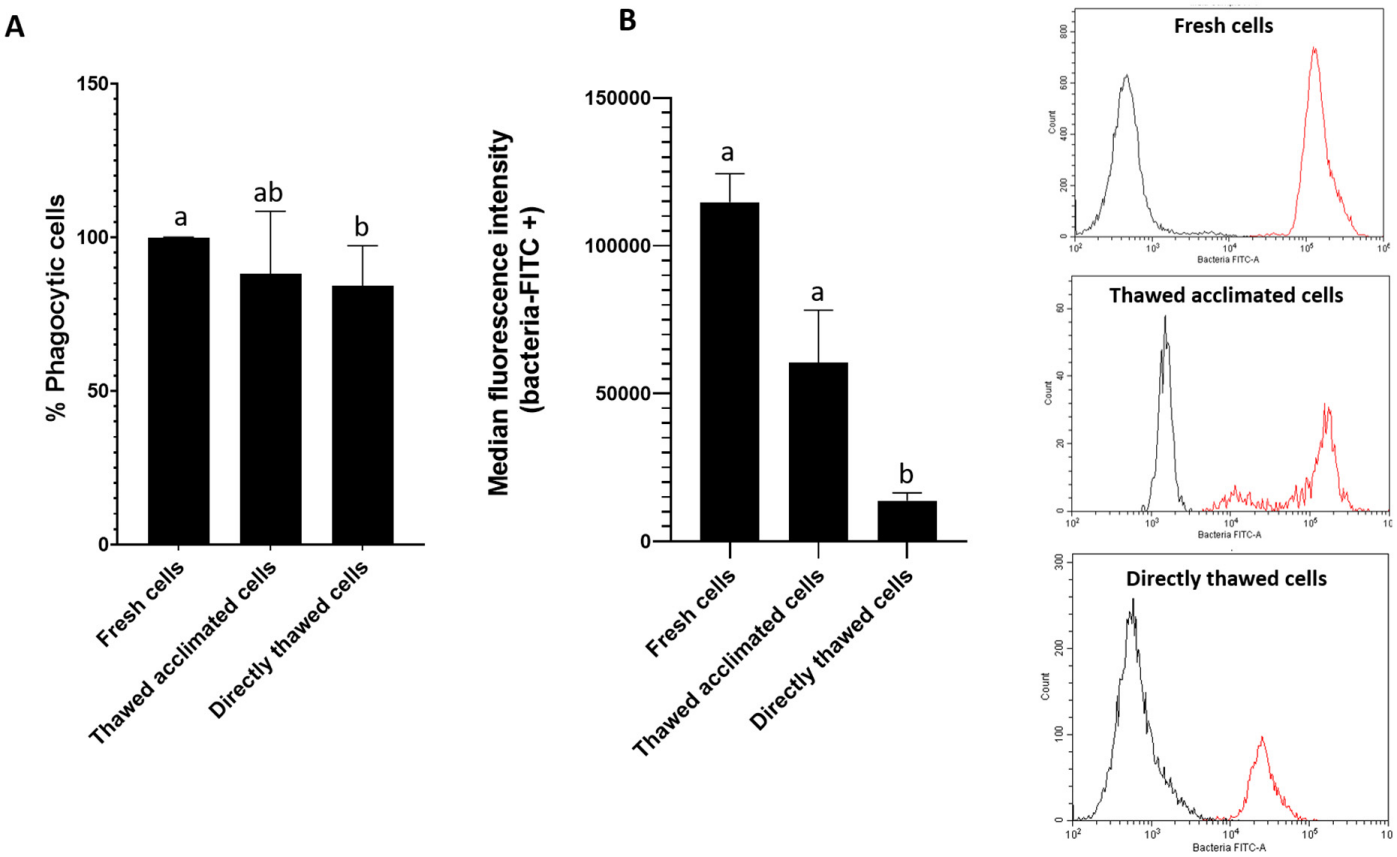
Flow cytometry assessment of cell (hemocytes) functionality by measuring cell phagocytic capacity. **(A)** Representation of the percentage of phagocytic cells for fresh, thawed acclimated, and directly thawed cells. **(B)** Median fluorescence intensity of the positive fluorescence population (bacteria + cell population) (left side), and fluorescence cytometry overlay (right side) showing the distribution of the total cell population for the three experimental groups (fresh, thawed acclimated, and directly thawed cells). The red curves represent positive fluorescence, while the black curves indicate negative fluorescence. Data in bar graphs represent the mean of values + SEM. Bars sharing the same letter do not present significant differences, while bars with different letters indicate statistically significant differences (p ≤ 0.05). Each letter corresponds to a specific statistical comparison among all groups.

The functional activity of the thawed cells was assessed by evaluating their capacity to phagocytize bacteria. Fresh cells presented a 100% phagocytic rate while values close to 90% were found for the thawed acclimated cells after the thawing process. The phagocytic activity measured immediately in directly thawed cells was a little lower (around 83%) (**Figure 5A**). These values indicate minimal variation among the three groups. However, despite comparable phagocytic rates in the three groups, the median of intensity fluorescence (**Figure 5B**), which reflects the quantity of phagocyted bacteria by each cell, showed variations among the three experimental groups. Specifically, thawed acclimated cells exhibited greater fluorescence intensity in comparison with the directly thawed cells, indicating higher bacterial intake per cell (**Figure 5B**).

The fluorescence peaks between cells in the presence (red lines) and in the absence (black lines) of fluorescent bacteria were clearly separated showing that fluorescence corresponded to bacteria uptake (**Figure 5B**). However, the intensity of the fluorescence peaks varied among the groups. Fresh cells peak was located around values of 10^5^ units of fluorescence whereas the peak for directly thawed cells was located in values of 10^4^ units. Interestingly, thawed acclimated cells displayed a double peak: one close to fresh cells (10^5^) and another similar to directly thawed cells (10^4^), suggesting that the thawed acclimated cells are gradually restore their functional activity following the acclimation period after thawing.

**Figure 6:**
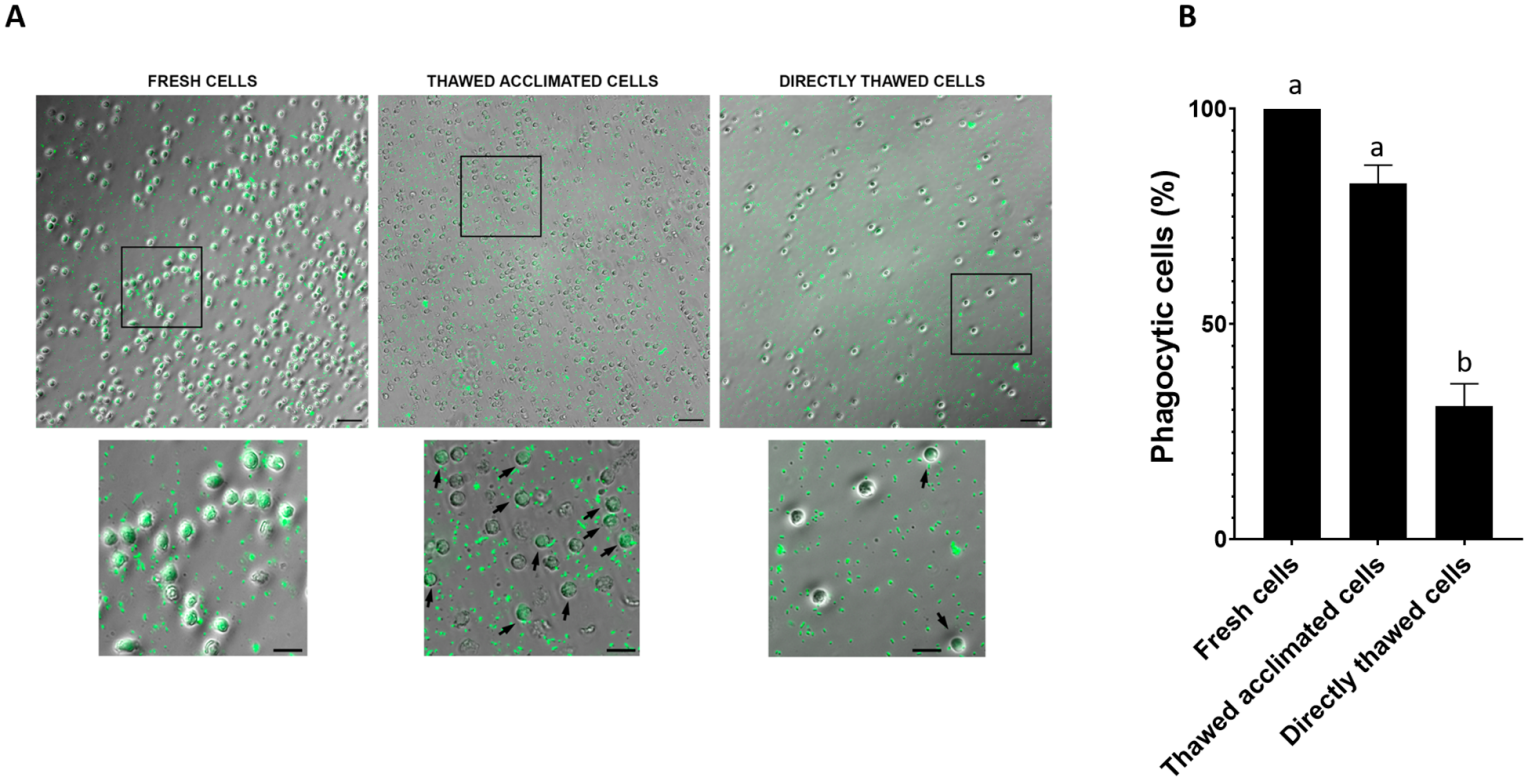
**(A)** Visualization of the phagocytic activity of fresh, thawed acclimated, and directly thawed cells (hemocytes) on FITC labeled bacteria (green points located inside the hemocytes). Positive phagocytic cells are pointed out with an arrow. Fresh cells were 100% positive and no arrows are indicated. For large images, scale bar: 50 µm; for close-ups, scale bar: 20 µm. **(B)** Image quantification of positive phagocytic cells (hemocytes). Data represent the mean of values + SEM. Bars sharing the same letter do not present significant differences, while bars with different letters indicate statistically significant differences (p ≤ 0.05). Each letter corresponds to a specific statistical comparison among all groups.

To further corroborate these findings, microscopic observations were conducted with FITC-labeled bacteria (**Figure 6A**). Both groups of thawed cells exhibited internalized fluorescent bacteria; however, thawed acclimated cells showed a higher efficiency in uptake. The percentage of phagocytosis in fresh cells was 100%, while in directly thawed cells the percentage of cells ingesting bacteria was reduced to 32.8%. Non-significant differences were found between fresh and thawed acclimated cells, which reached values close to 73% (72.7%) (**Figure 6B**). This observation supports the importance of an acclimation process in the recovery of functional cell activities post-thaw.

### 3.4 Cell viability

**Figure 7:**
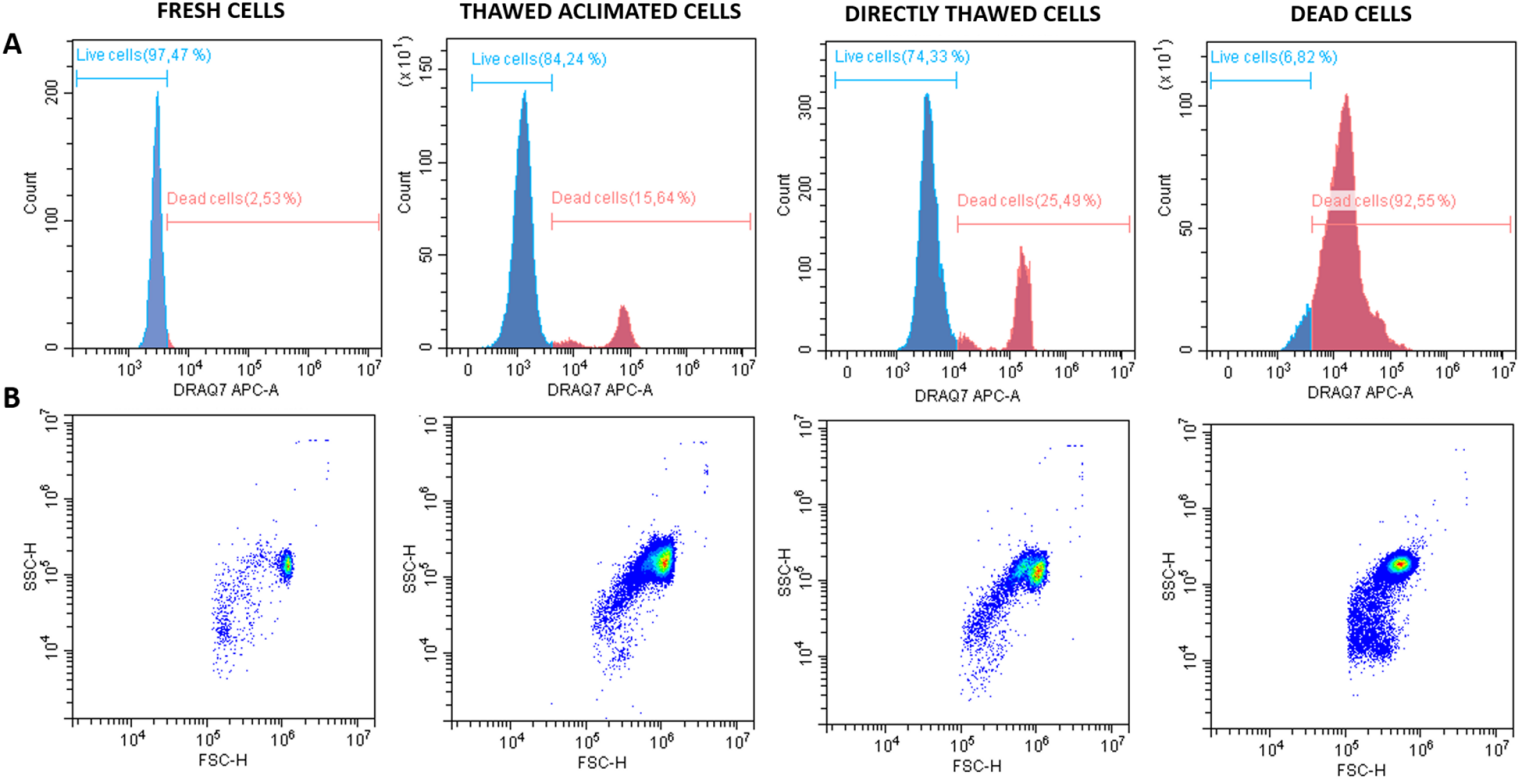
Histograms and dot plots of fresh, thawed acclimated, directly thawed, and dead cells (hemocytes) previously labeled with DRAQ-7 as death marker fluorophore. **(A)** Histograms represent the distribution of the counts on the fluorescence range. Blue curves correspond with live cells whereas red curves indicate the dead cells. **(B)** Dot plots represent the cell distribution according to size (FSC, forward scatter) on the X-axis, and granularity or complexity (SSC, side scatter) on the Y-axis for the four experimental analyzed groups.

**Figure 8:**
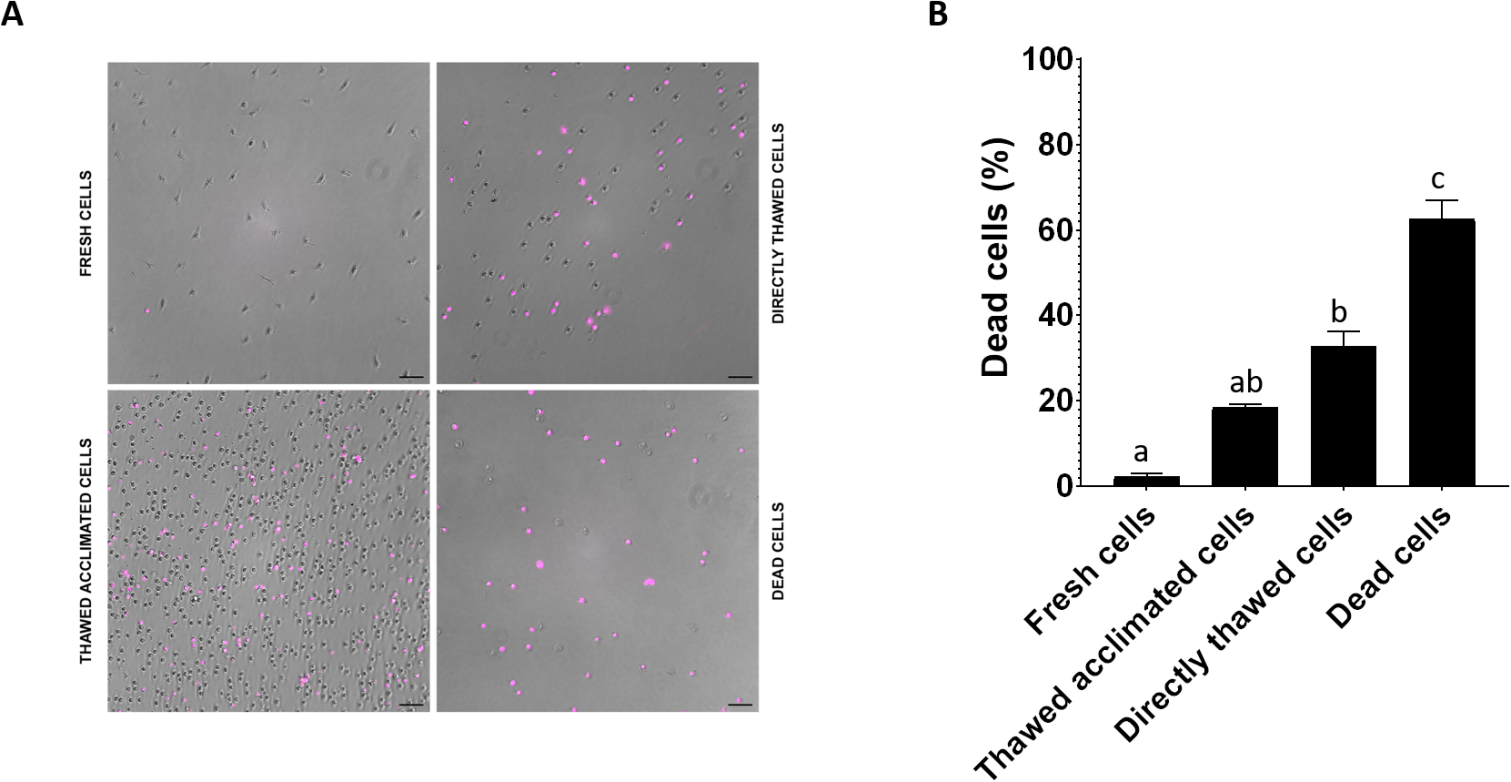
**(A)** Visualization under the optical microscope of fresh, thawed acclimated, directly thawed, and dead cells (hemocytes) previously incubated with DRAQ-7 as death marker fluorophore. Pink points correspond with death-positive cells. **(B)** Image quantification of positive DRAQ7 cells. Data represent the mean of values + SEM. Bars sharing the same letter do not present significant differences, while bars with different letters indicate statistically significant differences (p ≤ 0.05). Each letter corresponds to a specific statistical comparison among all groups.

The cell viability was assessed by flow cytometry across four experimental groups: fresh cells (positive control), thawed acclimated cells, directly thawed cells, and dead cells (negative control) with the frozen cells having been stored for two weeks before thawing (**Figure 7A**). Fresh cells presented viability close to 98%, while dead cells (induced by osmotic shock) showed viability below 7%. Directly thawed cells presented viability close to 75%, whereas those that underwent an acclimation process after thawing (thawed acclimated cells) showed viability nearing 85%. The directly thawed cells dot-plot revealed the presence of two cell populations corresponding with live cells on the right and membrane-compromised cells on the left side (with less light scatter), validating the importance of acclimation for maintaining viability (**Figure 7B**). Additional microscopic screening was also performed using a death-cell marker (DRAQ7) (**Figure 8A**) supporting the trends observed by flow cytometry in terms of the relationship between treatments (**Figure 8B**). No statistical differences were found between fresh and thawed acclimated cells, whereas significant differences were observed between fresh and directly thawed cells reinforcing the importance of the acclimation process.

### 3.5 Molecular assays

**Figure 9:**
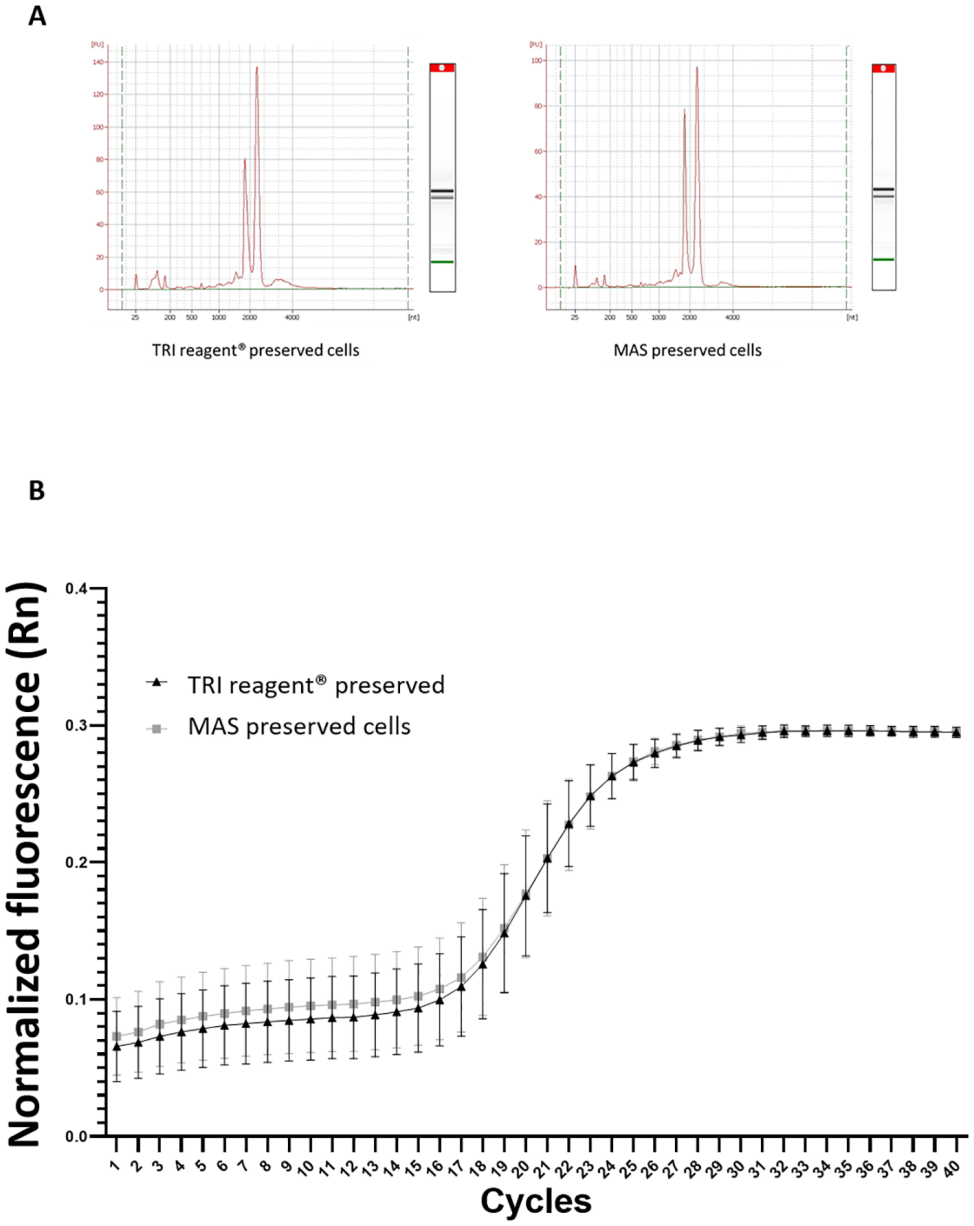
**(A)** RNA electropherogram profile obtained from an Agilent Bioanalyzer. The X-axis represents the migration time (seconds), while the Y-axis shows the fluorescence intensity, indicating the concentration of RNA present for samples cryopreserved in TRI Reagent^®^ and MAS medium. **(B)** Amplification curve from qPCR analysis of the housekeeping gene, ubiquitin. The graph shows the cycle number on the X-axis and the fluorescence intensity on the Y-axis. The triangles line represents the amplification curve for TRI reagent^®^ preserved cells (hemocytes), whereas the squares line indicates the amplification for MAS cryopreserved cells (hemocytes).

The RNA integrity profile (RIN) for samples of cells preserved in MAS medium supplemented with EG and for cells preserved in a known safety RNA medium: TRI Reagent^®^ was analyzed (**Figure 9A**). Both RIN profiles exhibited similar patterns with the expected 18S and 28S RNA peaks distinctly visible and no signs of degradation.

**Figure 10:**
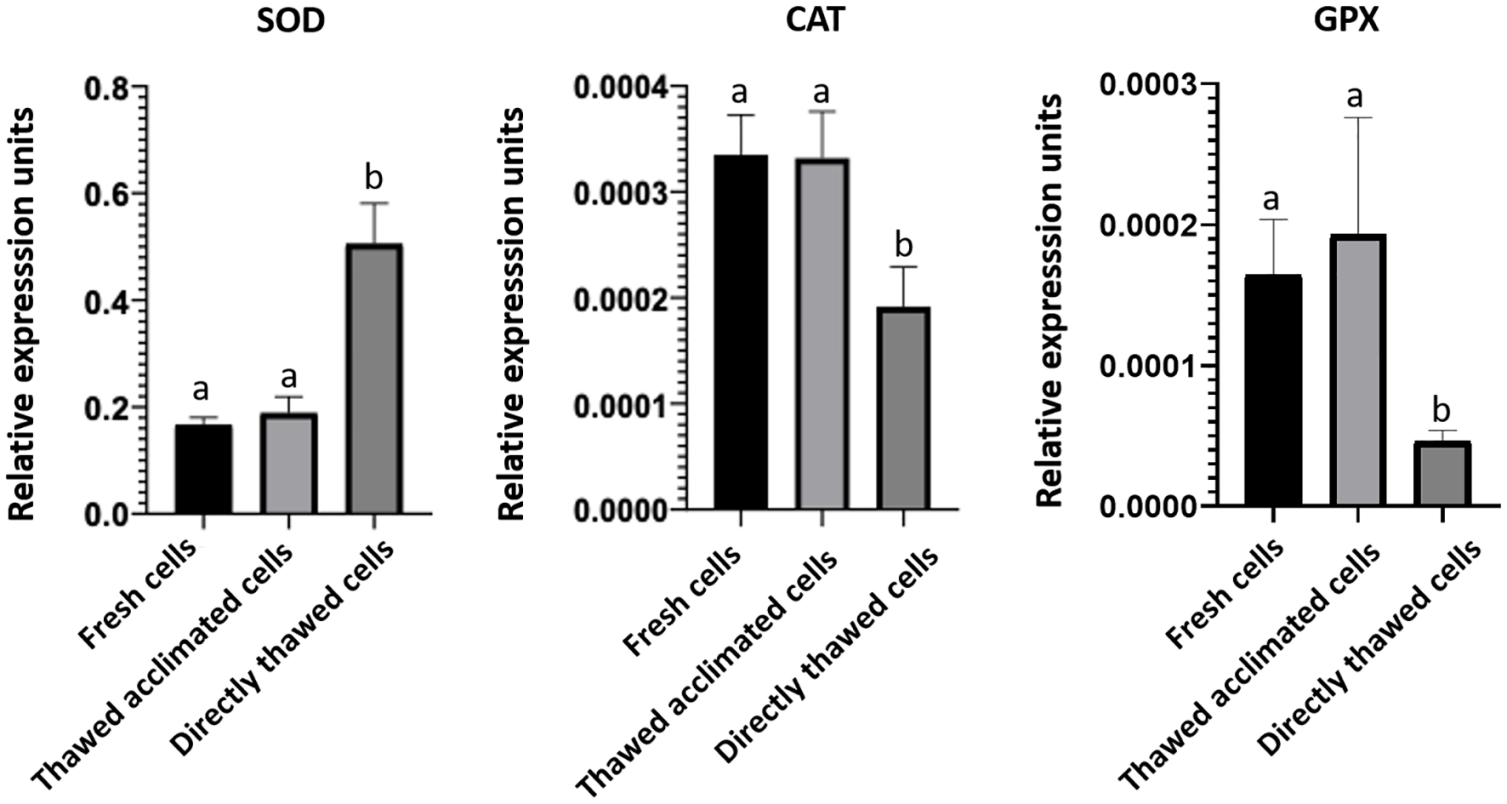
Expression of stress-related genes superoxide dismutase (SOD), catalase (CAT), and glutathione peroxidase (GPX). Bars represent the mean of the normalized expression of five biological replicates regarding to ubiquitin expression as housekeeping gene in fresh, thawed acclimated, and directly thawed cells (hemocytes). Bars sharing the same letter do not present significant differences, while bars with different letters indicate statistically significant differences (p ≤ 0.05). Each letter corresponds to a specific statistical comparison among all groups.

To assess any potential interference from the MAS medium in the gene expression detection —specifically during RNA isolation, reverse transcription, or qPCR— a housekeeping gene, ubiquitin, was amplified by qPCR (**Figure 9B**). The resulting amplification curves were nearly identical and even overlapped completely from cycle 20 onwards. These data, together with those obtained in the RIN, indicate that the MAS medium does not seem to interfere with RNA quality and integrity. The potential stress-inducing effects of the cryopreservation protocol were also analyzed across the three experimental groups of cells by evaluating the expression levels of the three stress-response genes. No significant differences in gene expression were detected between fresh and thawed acclimated cells. However, significant differences were found concerning directly thawed cells (**Figure 10**). These findings indicate that the acclimation of the cells after thawing helps restore cells to a state of fresh cells, particularly in terms of stress.

## 4 Discussion

The selection of the optimal preservation medium, cryoprotective agent CPA, and freezing/thawing protocol (including cooling and warming rates and temperatures) is highly specific for each cell type. Protocols effective for one-cell types might be completely unsuitable or even deleterious for another. For instance, glycerol is a widely used CPA for cryopreservation of sperm across different species (38) and for human red blood cells (39). However, many types of cells are poorly cryopreserved using glycerol as CPA due to cell-specific cytotoxicity, membrane permeability, and other unclear factors. The challenge lies in identifying specific protocols that can successfully preserve the functional and molecular characteristics of the cells under investigation. A complete cryopreservation formulation should include multiple components, such as mineral salts, buffering components, cell-specific nutrients, CPA agents, antioxidants, and scavengers (27). In this study, we evaluated four media, each with or without two different CPAs: EG or DMSO. Our findings revealed that the MAS medium produced the best viability after freezing and thawing, showing values close to 90% when supplemented with a CPA. No statistically significant differences were observed between DMSO and EG. Despite DMSO extensive popularity as CPA for various animal and vegetable cells, some studies suggest its possible role as a cell stress enhancer (40, 41). In addition, EG has shown potential as a proper effective CPA for other marine organisms, molluscs, in particular, yielding good survival rates, as well as low presence of larvae malformations (29, 42, 43). Although cryopreservation media must be tailored to each biological material, the lack of statistical differences found between the two tested CPAs and the literature support tipped the balance towards EG for use in the rest of the presented experiments.

However, it is worth noting that, in this specific case, either CPA could have been suitable. The MAS medium alone (without any CPA) was able to produce better viability values than other tested media even when supplemented with CPAs, indicating that the MAS formulation includes essential components for cell preservation. Specifically, MAS medium (16) contains glucose, which provides the energy required for basic maintenance and cell metabolism during cryopreservation and, like other sugars, offers antioxidant protection (44–46). It also includes trisodium citrate, which supplies mineral salts that provide an ionic environment that mimics natural extracellular conditions, maintaining the osmolarity and ionic balance. Additionally, trisodium citrate acts as a buffer, stabilizing pH during freezing and thawing, thereby protecting the cells from abrupt shifts in acidity or alkalinity that could damage them (47). Moreover, this molecule can also function as a scavenger by binding to metal ions such as calcium and magnesium, helping prevent precipitate formation and stabilize the solution (48). In the specific case of hemocytes, these cells as part of the defense mechanisms, tend to aggregate to capture microorganisms so to enhance the defensive response (49). These aggregates, while advantageous for the pathogens fighting, pose challenges during cryopreservation as the cells may not receive the cryoprotective medium equally. Furthermore, after thawing, individualization of the cells is necessary for future subsequent analyses, including single-cell analyses. MAS medium also contains citric acid, which plays a multifaceted role in cryopreservation, helping to maintain pH, sequestering metal ions, reducing oxidative stress, improving cell permeability, and stabilizing biomolecules (50, 51). The MAS formula is completed with EDTA, a chelating agent (52) and sodium chloride, which provides salts for keeping marine cells alive. No significant differences in the cell viability were found between MAS and SRS medium (33), which also contains mineral salts and chelating agents in its composition. However, microscopic observations before and after freezing/thawing (data not shown) revealed more debris and poorer cell morphology in SRS. Good viability was also observed when the cells were resuspended in OS. However, it is noteworthy that, to obtain the octopus serum, the hemolymph extraction was conducted at a 1:1 ratio with MAS medium as an anti-aggregant solution to prevent cell clotting. This dilution probably provided the OS with salts, chelating elements, and other optimal molecules that would facilitate the cryopreservation process. Despite this, viability values were significantly lower when using DMSO as CPA. Various other culture media were tested, although cell viability found was extremely low (data not shown). L-15 culture medium has been widely referenced in studies on primary cultures of marine cells (53, 54) and it was also satisfactorily used by Songco-Casey et al. (55), in their studies of *O. bimaculoides* visual system and by Styfhals et al. (56) in their analysis of the cell diversity of *O. vulgaris* brain. However, L-15 resulted in very high mortality for *O. vulgaris* immune cells in this study. These findings reinforce the importance of tailoring preservation methods to the specific needs of each cell type. Given these findings, we selected MAS medium supplemented with EG as the preferred medium for the remaining experiments.

In addition to the CPAs, effective cryopreservation also requires optimal cooling and warming rates to control cell dehydration during freezing, mitigate intracellular ice formation, and minimize the potential deleterious effect of CPAs during the thawing process (27). Based on prior findings obtained for marine molluscs, we used a cooling rate of -1°C/min (29) coupled with a progressive dilution of the freezing medium to minimize the CPA effect and prevent the cells from being damaged. Different studies have shown that progressive dilution during the thawing process is superior to fast dilution and thawing better preserves cell viability by allowing a gradual osmolarity equilibration and reducing the risk of osmotic shock, which can compromise cell membranes and overall cell survival (57, 58). Uhrig et al. (59) also highlighted in their review that optimized thawing protocols, including CPA dilution, significantly improve the post-thaw recovery and viability of induced pluripotent stem cells (iPSCs).

In our study, we compared “fast” (non-progressive) and “slow” (progressive dilution) thawing procedures for the four tested media. While previous studies documented lethal effects associated with the fast addition of CPA-free medium to a freshly thawed cell suspension, that effect was not significantly observed for those cells kept in MAS or OS media, further demonstrating the advantages of MAS medium in maintaining cell viability. For the other media tested, the choice of thawing method produced significant differences highlighting the importance of the used methodology. However, in this case, the MAS medium alone appears sufficiently robust as a cryopreservative medium.

Although cell viability gradually declined over time, MAS medium was able to maintain cells percentages above 70% after 15 weeks of storage at -80°C. It is well documented that extended storage time (even more in temperatures above the glass transition temperature) can affect cell viability. Several studies have examined how prolonged storage affects cell viability. Baust et al. (57) reported that CD34+ stem cells viability decreases with storage time, prolonged storage in liquid nitrogen led to a noticeable increase in early apoptosis, which negatively affects hematological recovery after transplantation. Li et al. (60) evaluated the impact of cryopreservation on porcine peripheral blood mononuclear cells (PBMC) and found that, while overall viability post-thaw was high, cell functionality —such as the ability to proliferate after antigenic stimulation— significantly declined. Our findings align with these studies, though it is important to emphasize that in our case, cell suspensions were not stored at the typical cryopreservation temperature of -196°C, the temperature of liquid nitrogen. In this study, cells were cryopreserved and then maintained at -80°C, a point well above the glass transition temperature. This temperature is not low enough to fully minimize biochemical reactions that can damage cells during prolonged storage and there is a risk of ice crystal formation that can perforate cell membranes and damage intracellular structures over time. Temperature fluctuations are minimal at -196°C, and chemical reactions and degradation processes are completely slowed down, helping preserve biomolecular integrity and better support physiological functions in stored cells (57, 60). The main objective of this work was to generate a larger window of time to carry out laboratory experiments that cannot be completed on the same day or that require shipping between labs while also aiming to reduce the number of animals used and the number of experimental procedures (injections, hemolymph extractions) performed on octopuses, thus promoting the application of the 3Rs principles. Therefore, we have only used storage at -80°C. The promising results obtained suggest that even better long-term preservation could be obtained at lower temperatures, such as -196°C. Further studies are needed to determine cell survival rates after storage in liquid nitrogen.

A key objective of this work was not only to preserve the cells in a viable state but also in a functionally active state. Given that preservation at -80°C does not offer the same long-term stability as storage in liquid nitrogen, evaluating the functional capacity of the cells was the next critical step. However, we wanted to compare not only the functionality of the cells immediately after thawing but also how was their phagocytic capacity after a period of acclimation to the new conditions. The ability to phagocytize is one of the tools used by the defense cells of these animals to fight against pathogens, as has been previously reported (11). Our results evidenced that thawed acclimated cells exhibited both a more flexible membrane (observed under an optical microscope) and an enhanced capacity to phagocytize fluorescent particles than directly thawed cells. These results are in agreement with previous studies, which have shown that an acclimation or resting period post-thaw can improve their functionality. Kuerten et al. (61) found that cryopreserved PBMCs benefited from a short resting period after thawing, which enhanced their immunogenicity, allowing the cells to recover from freezing/thawing stress and regain functionality. Similarly, Ortiz-Rodriguez et al. (62) demonstrated that post-thaw incubation of stallion sperm in a specific medium activated pro-survival pathways, enhanced mitochondrial function, and reduced stress, which are critical for maintaining cell functionality. In our case, we observed that thawed acclimated cells present higher phagocytosis rates, both in the percentage of phagocytic cells and in the median fluorescence intensity (results showed in **Figure 5**), indicating that there are more cells phagocytizing bacteria and that each cell ingested more bacteria when allowed an acclimation period. Visual evidence supported these results, where we observed that cells subjected to a post-thaw acclimation showed a fluorescence peak matching that of fresh cells and another matching that of directly thawed cells, suggesting that they are recovering their phagocytic capacities during this period of acclimation. Microscope images and the results derived from their quantification further illustrated that acclimated cells had a greater membrane flexibility probably correlating with a greater phagocytic capacity, as well as, higher levels of viability. Moreover, microscopic image analysis reinforced the flow cytometry results, showing that acclimated cells exhibited higher functionality compared to non-acclimated ones. Although the percentages of phagocytic cells differed between microscopy and flow cytometry assays due to the varying sensitivity of these techniques, the relationship among the three experimental groups remained consistent, further supporting the idea that cell acclimation helps cells recover their functional capacities. Flow cytometry analysis also revealed the presence of two cell populations (dead and alive cells) in the case of directly thawed cells, which are not present in the thawed acclimated cells.

In addition to ensuring that cryopreserved cells are viable and functional, another important objective of this work was to maintain cells in a state suitable for carrying out molecular assays such as sc-RNAseq analysis. As mentioned previously, this methodology requires starting from a population with a high viability percentage, where the cells are individualized and non-aggregated. Our results indicate these requirements are met, however, for a gene expression analysis it is essential to begin with high-quality RNA that is intact and free from degradation or contamination (63). To address this aspect, we aimed to analyze the RNA integrity of cells cryopreserved in MAS medium comparing it to TRI Reagent^®^, which has been proven as effective in maintaining RNA integrity during storage without significant adverse effects on gene expression (64). The lack of differences in RIN patterns between fresh cells directly frozen in TRI Reagent^®^ and cryopreserved cells in MAS medium supplemented with EG, demonstrates that the MAS+EG medium does not interfere on the RNA integrity during the subsequent RNA isolation by TRI Reagent^®^. The quality of total RNA populations can also be evaluated by measuring the expression of a housekeeping gene using reverse transcription-quantitative real-time polymerase chain reaction (RT-qPCR) (65). The efficiency of reverse transcription is contingent on the integrity of the initial RNA molecules and the results in the qPCR. The Ct values ​​obtained (indicating the number of cycles where the fluorescence obtained in the analyzed samples is higher than the established threshold) correlate with higher expression of the specific gene, meaning that greater RNA integrity results in lower Ct values. Our results revealed that Ct values for the housekeeping expression analyzed were similar for both tested media, suggesting that MAS media preserves RNA quality and integrity for common octopus hemocytes. To evaluate if the selected medium and thawing methodology could alter the basal status of the cells in terms of stress, we also analyzed the expression of a set of genes involved in the response against stressful conditions. Our results showed that thawed acclimated cells, alongside fresh cells, presented a similar pattern of expression concerning directly thawed cells. These differences suggest two different scenarios of cell behavior, with the post-thawing acclimation process seemingly promoting cell recovery, bringing thawed acclimated cells to a similar basal status to that of fresh cells. Notably, the expression profile of SOD was different from CAT and GPX across the three cell-analyzed groups. These differences in the regulation of the anti-oxidant enzymes between fresh, thawed acclimated, and directly thawed cells may reflect a differential response to the stress caused by the freezing-thawing process, especially in the absence of a recovery period. After thawing, cells tend to undergo an increase in ROS production, usually due to mitochondrial damage and a fast cell re-oxygenation (66, 67), which could explain the increased SOD expression in directly thawed cells in comparison with the other experimental cell groups. This enzyme is the first line of defense against anion superoxide (O_2_^-^) (68), a primary ROS generated as a by-product of altered mitochondrial metabolism. The observed increase in SOD suggests a cell response to counteract the excess of ROS generated in the freezing-thawing context. Importantly, the acclimation process reduced SOD expression to levels comparable to those of fresh cells. Conversely, the expression of CAT and GPX, enzymes responsible for detoxifying hydrogen peroxide (H_2_O_2_) produced by SOD, was lower in directly thawed cells than in fresh or thawed acclimated cells. Using these expression levels as a stress marker, the CAT and GPX expression results observed for directly thawed cells are significantly striking.

In our opinion, this seemingly non-stressful status observed in this set of cells is not such, since these levels of expression could be due to a deficiency in the response, which could be produced by a disequilibrium in the transcriptional regulation (69, 70). For instance, it has been demonstrated that cell stress produced by the freezing-thawing process could damage cell structures and deplete the reduced glutathione levels (GSH), an essential cofactor for the GPX activity, also affecting the maintenance of a balanced redox environment and producing a lower expression of these enzymes (71). In addition, the O_2_- is a small and highly reactive molecule (72), which is quickly transformed in H_2_O_2_ by SOD. However, the H_2_O_2_ is more stable than O_2_- (73) and can be accumulated, whereby CAT and GPX need to deal with higher concentrations of substrate, which can be lethal if they are not detoxified quickly.

These differential expression patterns may reflect cell energy prioritization, with cells initially favoring protection against O_2_- via SOD activity, while delaying the production of H_2_O_2_ detoxifying enzymes during the early stages of cold-induced damage (74). These results indicate that the process of acclimation not only enhances cell viability and functionality, but also significantly mitigates the cell stress provoked by the freezing-thawing process.

In conclusion, the data presented in this paper introduce a novel methodology for cryopreservation of the primary immune system cells of *O. vulgaris*, a vital model species for numerous biological studies and an excellent candidate for aquaculture diversification. The reported data demonstrate the viability, functionality, and molecular integrity of cryopreserved octopus cells, making these cells suitable now for multiple new assays. The successful cryopreservation of these cells represents a significant advancement that will facilitate the development of techniques previously deemed impossible, enabling further exploration of the defense mechanisms of a highly evolved species strategically positioned in the evolutionary landscape.

## Data Availability

The original contributions presented in the study are included in the article/supplementary material. Further inquiries can be directed to the corresponding author.
